# Feasibility and early clinical assessment of flattening filter free (FFF) based stereotactic body radiotherapy (SBRT) treatments

**DOI:** 10.1186/1748-717X-6-113

**Published:** 2011-09-12

**Authors:** Marta Scorsetti, Filippo Alongi, Simona Castiglioni, Alessandro Clivio, Antonella Fogliata, Francesca Lobefalo, Pietro Mancosu, Pierina Navarria, Valentina Palumbo, Chiara Pellegrini, Sara Pentimalli, Giacomo Reggiori, Anna M Ascolese, Antonella Roggio, Stefano Arcangeli, Angelo Tozzi, Eugenio Vanetti, Luca Cozzi

**Affiliations:** 1 Radiotherapy and radiosurgery, Humanitas Cancer Center, Istituto Clinico Humanitas, Rozzano (Milano), Italy; 2 Oncology Institute of Southern Switzerland, Bellinzona, Switzerland

**Keywords:** Flattening Filter Free, SBRT, RapidArc, TrueBeam

## Abstract

**Purpose:**

To test feasibility and safety of clinical usage of Flattening Filter Free (FFF) beams for delivering ablative stereotactic body radiation therapy (SBRT) doses to various tumor sites, by means of Varian TrueBeam™ (Varian Medical Systems).

**Methods and Materials:**

Seventy patients were treated with SBRT and FFF: 51 lesions were in the thorax (48 patients),10 in the liver, 9 in isolated abdominal lymph node, adrenal gland or pancreas. Doses ranged from 32 to 75 Gy, depending on the anatomical site and the volume of the lesion to irradiate. Lung lesions were treated with cumulative doses of 32 or 48 Gy, delivered in 4 consecutive fractions. The liver patients were treated in 3 fractions with total dose of 75 Gy. The isolated lymph nodes were irradiated in 6 fractions with doses of 45 Gy. The inclusion criteria were the presence of isolated node, or few lymph nodes in the same lymph node region, in absence of other active sites of cancer disease before the SBRT treatment.

**Results:**

All 70 patients completed the treatment. The minimum follow-up was 3 months. Six cases of acute toxicities were recorded (2 Grade2 and 2 Grade3 in lung and 2 Grade2 in abdomen). No patient experienced acute toxicity greater than Grade3. No other types or grades of toxicities were observed at clinical evaluation visits.

**Conclusions:**

This study showed that, with respect to acute toxicity, SBRT with FFF beams showed to be a feasible technique in 70 consecutive patients with various primary and metastatic lesions in the body.

## Introduction

In case of tumors at early stage, or in case of isolated small metastases, stereotactic body radiation therapy (SBRT) has proved to be a safe and feasible treatment approach, as demonstrated by the tumor response and local control rates in selected series [1].

Improvements in screening intensification and in management techniques have reached high levels of accuracy so that it is possible to detect tumors at rather early stages. The paradigm of the usefulness of SBRT in localized primary tumors is early non small cell lung cancer (NSCLC). Highly focused doses of 60-66 in three fractions with SBRT to NSCLC in early stages, achieve an actuarial 2-year local control of 95%[2]. The position of the lesion is a limitation in dose escalation: although less than 20% of patients showed high-grade toxicity, toxicity greater than grade 3 were more frequent in patients with tumors proximal to the bronchial tree or central chest region [3]. In a retrospective review, Onishi et al: analyzed a large number of SBRT treatments from a Japanese multi-institutional database showing that SBRT is safe and promising as a radical treatment for operable Stage I NSCLC [4]. When the effective biologic dose was greater than 100 Gy, the survival rate was higher.

Optimization of systemic cytotoxic chemotherapy schemes and tailored drugs are improving significantly survival of cancer patients. In this scenario, it is possible to perform aggressive treatment of oligometastatic disease with curative-intent [5].

Promising studies, are exploring the safety and feasibility of SBRT in abdominal and pelvic sites [6-13]. Rusthoven showed that, in patients with metastatic liver lesions, it is possible to deliver safely 60 Gy, in three fractions [13].

As a consequence, large groups of patients with primary or metastatic lesions can deserve SBRT, as curative approach and improvements in precision and accuracy are advisable to allow safe prescription of more ablative doses.

Recently, two new technological platforms have been made available to clinical practice. Firstly, Volumetric Modulated Arc Therapy (VMAT) in its RapidArc^® ^format, allowed to reduce significantly the time needed to deliver complex intensity modulated plans, allowing to treat hypofractionated regimes within few minutes [14-17]). Secondly, there has been increasing attention into the clinical use of linear accelerators (LINAC) with photon beams generated without usage of the flattening filter [18-24]. It seems possible to expect a reduction of out-of-field dose when flattening filter free (FFF) beams are used. This is mainly due to reduced head scatter and residual electron contamination. FFF beams should therefore lead to reduced peripheral doses and patients may benefit by decreased exposure of normal tissue to scattered doses outside the field. Removal of the flattening filter implies also the possibility to deliver treatments with higher dose rates, up to factor 4 at 10 MV, and with a much higher dose per pulse. This, beside further improving time efficiency for delivery, might have subsequent potential radiobiology implications, now still unclear and deserving dedicated investigations. While research in the physics domain for FFF beams is increasing, there are very few clinical data where FFF beams are applied in clinical practice, particularly in SBRT treatments.

Over the last few years the clinical introduction of RapidArc^® ^in SBRT was explored and, to test the feasibility and safety of combining this technique with FFF beams, a group of patients was treated with ablative SBRT doses to various anatomical tumor sites, by means of the recently introduced Varian TrueBeam™ system (Varian Medical Systems, Palo Alto, CA, USA) [15,16,24,25]. The evaluation of the role of FFF beams in reducing involvement of organs at risk while preserving adequate target coverage is not aim of the present study.

## Materials and methods

### Population of study

This is a prospective study and it was approved by the Institution. Between September and December 2010, a total of 123 patients were treated with the TrueBeam™ newly installed. Seventy out of 123, were treated with SBRT and FFF beams (SBRT-FFF group). In this group, 51 lesions were in the thorax (48 patients) and 19 in the abdomen: 10 liver, 4 isolated abdominal lymphnode metastases, 3 adrenal gland, 2 pancreas. Table 1 summarizes demographic data. Patients will be stratified into three groups: lung, abdominal and liver cases.

**Table 1:** Main characteristics of patients cohort

Gender	
Male	46 (66%)

Female	24 (34%)

**Age (y)**	

Median	65

Range	39-89

**Primary site**	

Lung	34(48%)

Colon	10(14%)

Breast	2(3%)

Pancreas	2 (3%)

Uterus	1 (1,5%)

Sarcoma	6 (9%)

Stomach	3 (4, %)

Prostate	1(1,5%)

Liver	4(6%)

Endometrial	1(1,5%)

Melanoma	1(1,5%)

Unknown	5 (7%)

### Protocols: inclusion criteria and dose prescription

Prescription doses ranged from 32 to 75 Gy (mean dose to target volume), depending on the site and on the volume of the lesions. Good performance status and good compliance to radiation treatment were requested in all patients. Lung lesions (primary early NSCLC and oligometastatic cases) were treated with cumulative doses of 32 or 48 Gy, delivered in 4 consecutive daily fractions. The dose of 32 Gy was prescribed to lesions located centrally or at a distance < 2 cm from the carena and/or to lesion with maximum diameter > 3 cm. In the remaining cases, a dose of 48 Gy was prescribed.

The ten liver patients, were treated for metastatic lesions in 3 fractions up to 75 Gy. Eligible patients met these criteria: inoperable or medically unsuitable for resection, maximum tumour diameter < 6 cm, one to three hepatic lesions. The 4 isolated lymph nodes, the adrenal gland and pancreas cases were irradiated in 6 fractions up to 45 Gy. The inclusion criteria were the presence of isolated or few lymph nodes in the same lymph node region, in absence of other active sites of cancer disease.

CT scans for planning were acquired for all patients positioned supine with their arms above the head and immobilized by means of a thermoplastic body mask (including a styrofoam block for abdominal compression to minimize internal organ motion in abdominal cases). Contrast free and Contrast-enhanced planning CT scans were acquired in free breathing mode at 3 mm slice thickness.

The clinical target volume (CTV) included macroscopic and microscopic disease on CT as well as on PET if available. The planning target volume (PTV) was generated by taking into account both the internal margin (IM) and the set-up margin (SM). IM depends on intra- and inter-fraction organ motion (expected to be not significant in a short course of radiation for retroperitoneal nodes adjacent to spine and large vessels while it may be relevant for lung, pancreatic and liver tumours) [26-30]. Since SM was minimised by the cone-beam CT (CBCT) verification of set up variations, the overall standardised CTV-PTV margin was prescribed as 6-15 mm in the cranial-caudal axis and 3-8 mm in the anterior-posterior and lateral axes. For 70% of the lung patients, 4 DCT retrospective scans were acquired for planning purpose and PTV was defined using smaller margins, i.e. 5 mm isotropic in all directions.

Planning objectives to target coverage aimed to cover PTV with 95% of the prescribed dose (reduced to 67% for few liver metastases cases whenever it was impossible to respect dose constraints to organs at risk). The main organs at risk (OAR) considered, depending on the treatment site, were: lungs, oesophagus, spinal cord, heart, kidneys, stomach, duodenum, small bowel and liver. Stomach, duodenum and small bowel were contoured when appropriate. For OARs, plans were required to meet explicit objectives as follows: Spinal cord: D_0.1 cm3 _< 18 Gy (dose lower than 18 Gy tot a volume of 0.1 cm^3^); V_15 Gy _< 35% for both kidneys (less than 35% of the volume receiving 15 Gy), V_36 Gy _< 3% for stomach and small bowel, V_15 Gy _< (total liver volume minus 700 cm^3^, i.e. at least 700 cm^3 ^of liver should receive less than 15 Gy) for liver. In addition D_0.5 cm3 _< 30 Gy for stomach and small bowel was considered as a secondary objective. For lungs V_5 Gy _< 30%, V_10 Gy _< 12% V_20 Gy _< 10%. For heart and for oesophagus no explicit planning objectives were applied.

### SBRT procedure

TrueBeam™ is a new LINAC designed to deliver flattened, as well as flattening filter-free (FFF) photon beams. In TrueBeam™, many key elements including the waveguide system, carousel assembly, beam generation, and monitoring control system differ from the preceding LINAC series as described in [24].

All patients were treated with RapidArc^® ^with 6 (11 cases) or 10 MV (59 cases) FFF beams. Energy selection was based on achievable plan quality. The maximum dose rate enabled for FFF beams was 1400 MU/min for 6 MV and 2400 MU/min for 10 MV [30]. RapidArc^® ^plans were individually designed using full or partial single or multiple arcs chosen to obtain the best adherence to planning objectives for each patient. All dose distributions were computed with the Analytical Anisotropic Algorithm (AAA, version 8.9 [31]) implemented in the Eclipse planning system with a calculation grid resolution of 2.5 mm.

A feature of TrueBeam™, applied to all the patients in the study, is the so-called 'jaws tracking' mode. In this mode, the main jaws of the LINAC are dynamically moved by the control system to the minimum aperture needed to cover target projection and to maximize organs at risk sparing at each gantry projection.

Treatment was delivered in 3 to 6 consecutive working days, with the patient on a 3-hour fast to avoid gross displacement of stomach and bowel. Treatment delivery included stereotactic frame localization and CBCT in the first session aiming at a preliminary isocentre positioning while for following fractions, patient set-up was realised by means of CBCT image guidance with, eventually, on-line couch adjustment at each fraction. Image matching was performed on bones and, when visible, on tumors and other soft tissue structures (e.g. main blood vessels).

### Evaluation of dosimetric and technical data

For each group of patients, technical parameters of delivery were scored in terms of number of arcs, total number of monitor units (MU), monitor units per Gy (MU/Gy), total beam on time. Dosimetric quality of treatments was measured from dose volume histogram (DVH) analysis. For PTV, the target coverage (mean, D_1%_, D_95%_, V_67%_, V_80%_, V_95%_, V_107%_), the homogeneity (Standard Deviation) and the conformity for PTV (CI_95%_) were reported. CI was defined as the ratio between the volume of patient irradiated at 95% of the prescribed dose and the PTV volume [32]. For OARs, the mean dose, the maximum dose (D_xcm3_) and appropriate values of V_xGy _(volume receiving at least xGy) were scored.

### Evaluation of clinical data

Clinical evaluations were planned on first day of treatment, before SBRT-FFF session (visit 0); visit 1 during the course of the treatment; visit 2 at the end of the last session; visit 3 within 60-90 days from the end of the treatment. Unscheduled visits could be performed if necessary.

Acute radiation induced toxicities were scored according to NCI Common Terminology Criteria for Adverse Events (CTCAE version 3.0) [33].

A first assessment of treatment outcome, although obviously very early, was performed at first and second follow up visits and will be reported in terms of degree of response.

## Results

### Dosimetric and technical data

**Figure 1:**
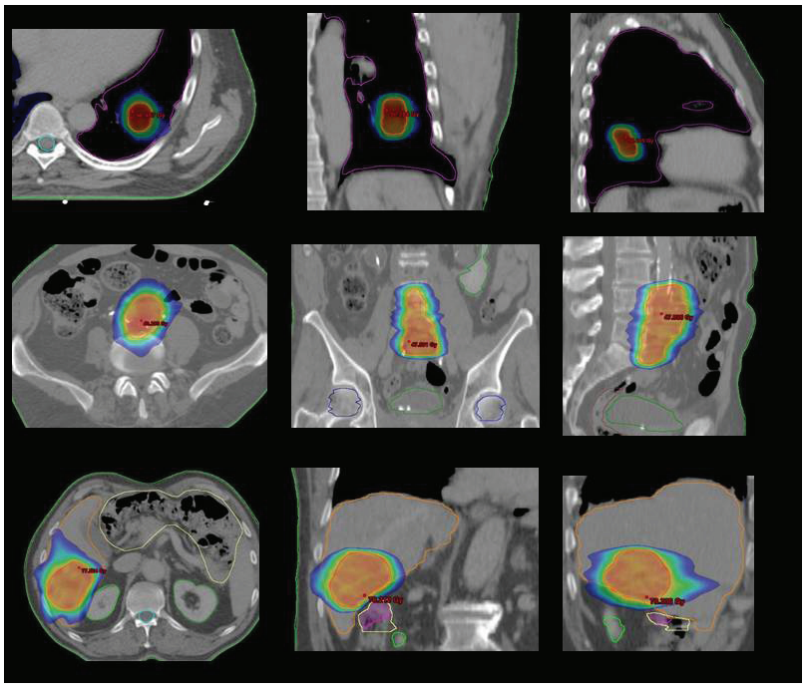
**Examples of dose distributions for the three groups of patients**. Colourwash scale is from 20 to 50 Gy for the lung and the abdominal cases and from 35 to 80 Gy for the liver case.

**Figure 2:**
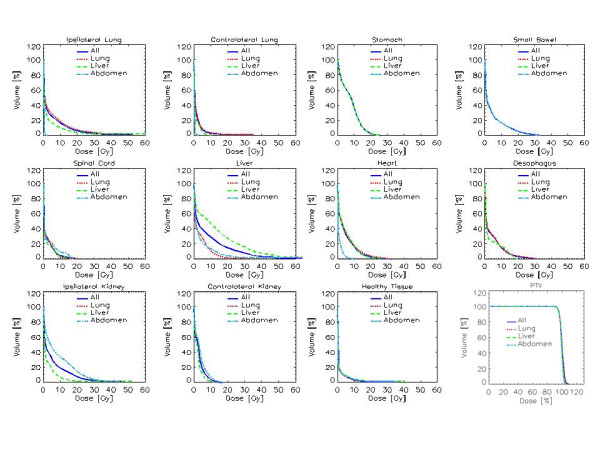
**Average cumulative DVH for OARs and PTV for all patients and stratified in the three groups**.

Figure 1 illustrates examples of dose distributions of lung, abdominal and liver patients with display of an axial, sagittal and coronal plane. Figure 2 present average cumulative dose volume histograms for PTV and all organs at risk for the entire cohort of patients and for the three sub groups.

Table 2 shows results from DVH analysis for target volumes, stratified according to localization. Table 3 contains the results for OARs.

**Table 2:** Summary of dose volume histogram analysis for PTV and healthy tissue

Parameter	All	Lung	Liver	Abdomen
**PTV**				

**Volume [cm^3^]**	78 ± 78	55 ± 55	146 ± 116	115 ± 82

**Mean dose [%]**	101.0 ± 2.9	101.2 ± 1.0	100.6 ± 1.6	100.3 ± 0.5

**St. Dev. [%]**	2.7 ± 0.6	2.9 ± 0.5	2.3 ± 0.8	2.1 ± 0.6

**D_1% _[%]**	106.0 ± 1.8	106.8 ± 1.5	104.3 ± 1.6	104.4 ± 1.4

**D_95% _[%]**	96.2 ± 1.0	96.1 ± 0.9	96.2 ± 1.5	96.4 ± 0.8

**V_95% _[%]**	97.3 ± 1.9	97.1 ± 1.7	97.2 ± 3.0	97.9 ± 1.5

**V_107 _[%]**	1.5 ± 5.1	2.1 ± 6.0	0.1 ± 0.2	0.0 ± 0.1

**Healthy tissue**				

**Volume [cm^3^]**	30660 ± 8928	29283 ± 7493	36232 ± 13301	31510 ± 8805

**Mean [Gy]**	1.2 ± 0.7	1.1 ± 0.8	1.4 ± 0.3	1.3 ± 0.6

**V_10 Gy _[%]**	3.3 ± 2.7	3.0 ± 3.0	4.0 ± 1.3	3.9 ± 2.4

**CI_95%_**	1.2 ± 0.3	1.3 ± 0.3	1.2 ± 0.3	1.2 ± 0.3

Dose Int. [Gy*cm^3^*10^4^]	3.5 ± 2.1	3.1 ± 2.0	5.0 ± 2.1	4.1 ± 1.8

V_xGy_: volume receiving at least × Gy. D_x%_: dose received by at least x% of the volume. CI: Conformity Index

**Table 3:** Summary of dose volume histogram analysis for organs at risk

Parameter	All	Lung	Liver	Abdomen
	**Ipsilateral Lung**			

**Volume [cm^3^]**	1814.5 ± 619.0	1861.9 ± 610.5	1686.4 ± 797.2	1407.2 ± 76.1

**Mean [Gy]**	4.4 ± 3.0	4.9 ± 2.8	3.1 ± 3.1	0.2 ± 0.0

**V_5 Gy _[%]**	24.6 ± 18.3	27.6 ± 17.6	16.1 ± 17.0	0.0 ± 0.0

**V_10 Gy _[%]**	15.5 ± 12.6	17.4 ± 12.3	9.5 ± 11.8	0.0 ± 0.0

**V_20 Gy _[%]**	5.8 ± 4.8	6.6 ± 4.6	3.3 ± 5.4	0.0 ± 0.0

	**Contralateral Lung**			

**Volume [cm^3^]**	1703 ± 629	1774 ± 615	1352 ± 765	1484 ± 335

**Mean [Gy]**	1.6 ± 2.1	1.8 ± 2.2	1.0 ± 0.6	0.2 ± 0.1

**V_5 Gy _[%]**	7.0 ± 14.3	8.0 ± 15.7	3.8 ± 3.5	0.0 ± 0.1

**V_10 Gy _[%]**	2.7 ± 8.3	3.2 ± 9.2	0.1 ± 0.3	0.0 ± 0.0

**V_20 Gy _[%]**	0.9 ± 2.9	1.1 ± 3.2	0.0 ± 0.0	0.0 ± 0.0

	**Spinal cord**			

**Volume [cm^3^]**	65 ± 35	61 ± 36	76 ± 33	78 ± 28

**D_0.1 cm3 _[Gy]**	9.9 ± 5.5	10.2 ± 6.1	9.4 ± 3.1	8.8 ± 4.9

**D_1% _[Gy]**	9.0 ± 4.5	9.1 ± 4.8	8.8 ± 3.2	8.4 ± 4.8

	**Liver**			

**Volume [cm^3^]**	1473 ± 409	1735 ± 606	1546 ± 434	1266 ± 193

**Mean [Gy]**	8.0 ± 5.8	3.2 ± 2.6	12.9 ± 3.7	3.8 ± 3.6

**V_15 Gy _[%]**	18.8 ± 17.1	3.2 ± 2.2	33.7 ± 10.7	6.2 ± 8.9

	**Ipsilateral Kidney**			

**Volume [cm^3^]**	137 ± 38	-	134 ± 42	141 ± 38

**Mean**	5.0 ± 5.3	-	2.5 ± 2.4	7.8 ± 6.5

**V_15 Gy _[%]**	11.5 ± 20.0	-	2.2 ± 3.6	22.4 ± 26.0

	**Contralateral kidney**			

**Volume [cm^3^]**	139 ± 35	-	126 ± 37	151 ± 30

**Mean [Gy]**	2.7 ± 2.1	-	2.0 ± 1.1	3.4 ± 2.7

**V_15 Gy _[%]**	0.2 ± 0.7	-	0.0 ± 0.0	0.4 ± 1.0

	**Stomach**			

**Volume [cm^3^]**	104 ± 40	-	104 ± 40	-

**Mean [Gy]**	7.3 ± 2.8	-	7.3 ± 2.8	-

**V_36 Gy _[%]**	0.0 ± 0.0	-	0.0 ± 0.0	-

**D_0.5 cm3 _[Gy]**	20.6 ± 3.6	-	20.6 ± 3.6	-

	**Small Bowell**			

**Volume [cm^3^]**	1255 ± 569	-	-	1255 ± 569

**Mean [Gy]**	4.1 ± 4.4	-	-	4.1 ± 4.4

**V_36 Gy _[%]**	0.0 ± 0.0	-	-	0.0 ± 0.0

**D_0.5 cm3 _[Gy]**	24.3 ± 13.0	-	-	24.3 ± 13.0

	**Heart**			

**Volume [cm^3^]**	624 ± 190	629 ± 174	676 ± 298	365 ± 0.0

**Mean [Gy]**	4.3 ± 4.0	4.5 ± 4.2	4.3 ± 3.3	0.9 ± -0.0

**D_1% _[Gy]**	15.3 ± 9.6	15.9 ± 9.7	14.9 ± 10.7	6.1 ± -0.0

	**Oesophagus**			

**Volume [cm^3^]**	39 ± 43	32 ± 34	91 ± 86	-

**Mean [Gy]**	3.8 ± 2.9	3.8 ± 3.0	3.3 ± 3.7	-

**D_1% _[Gy]**	13.6 ± 7.9	13.9 ± 8.0	10.8 ± 10.2	-

V_xGy_: volume receiving at least × Gy. D_x%_: dose received by at least x% of the volume.

Table 4 summarizes the technical delivery parameters.

**Table 4:** Main Technical features of delivered treatments

	Mean ± SD	Range
**MU**	2780 ± 1493	[629÷6734]
**MU/Gy**	283.6 ± 79.7	[164.1÷551.5]
**MU/arc**	1955 ± 1312	[315÷6099]
**MU/deg**	7.6 ± 4.3	[1.1÷17.2]
**DR [MU/min]**	1541 ± 621	[327÷2400]
**Gantry speed [deg/s]**	4.4 ± 1.6	[1.4÷6.0]
**CP aperture [cm]**	1.7 ± 0.8	[0.2÷4.4]
**CP area [cm^2^]**	14.5 ± 10.4	[3.6÷55.2]
**Arc length [deg]**	258.5 ± 70.5	[158÷358]
**Beam on time [min]**	1.7 ± 0.7	[0.9÷4.4]

See Additional figure 1 and 2

Values are reported as averages over all patients, arcs and/or control points

Target coverage (D_95%_) and homogeneity were similar to those of abdomen SBRT treatments, published by our group, characterized by high degree of conformality and modest target overdosage (V_107%_)[15]. For OARs, it was possible to respect planning objectives in most of the cases, also in the case of ipsilateral lung with a mean dose smaller than 5 Gy and V_5 Gy _< 30%.

Analysis of the technical delivery parameters showed that the availability of extended dose rate with FFF beams was fully exploited by the RapidArc^® ^technique with an average DR of 1500 and range spanning from about 300 MU/min to a maximum of 2400 MU/min (leading to a relatively wide range of MU/deg from about 1 to about 17). As a consequence, although the dose per fraction reached 25 Gy, the beam on time, was kept very small with a range from < 1 min to 5 min.

### Clinical Data

All 70 SBRT-FFF patients completed the treatment, as programmed. The minimum follow-up was 3 months. Six cases of acute toxicities were recorded (2 Grade 2 and 2 Grade 3 in lung and 2 Grade 2 in abdomen). No patient experienced acute toxicity greater than Grade 3. No other types or grades of toxicities were experienced at clinical evaluations.

In 55 out of 70, early clinical outcome was assessable at first diagnostic evaluation with PET and/or CT: complete response was achieved in 10 patients, partial response was in 26, and in 13 disease remained stable. Progression was found in 6 irradiated lesions.

## Discussion

In the current study we report on the treatment of a group of patients undergoing SBRT with RapidArc^® ^technique in combination with flattening filter free photon beams with the new TrueBeam™ LINAC. The rationale of the use of FFF beams for delivering SBRT doses, is the potential possibility to deliver high ablative doses faster and more precisely, due to decreased out-of-field dose and to increased dose rate removing flattening filter [24]. In addition, the time factor linked to the very high dose rates available, suggests that FFF beams might be of interest also in the case of respiratory gated treatments where the trade-off of low duty cycle might be efficiently compensated. Another possible and relatively obvious immediate advantage of high dose rate of FFF beams is linked to the potential reduction of intra-fraction motion, due to the reduction of total session treatment time. All these aspects were not directly addressed in the present study. The present study was limited to a more primordial aim: the demonstration of the clinical feasibility of SBRT treatments with FFF beams, the demonstration of short term safety of these (in terms of acute toxicity) and the investigation of dosimetric and technical features of the treatments.

The objectives of this study were to evaluate feasibility and safety of SBRT with FFF beams. Although, in hypofractionated treatments performed the most significant expected complications are usually the late effects, it is remarkable that acute toxicity recorded in our population of study was mild, confirming the feasibility and safety of the clinical use of FFF beams in SBRT patients, which are the end points of the current evaluation in the study.

It is established that late effects are frequently related to the intensity of acute toxicity and based only on this statement we can expect promising long term tolerability. On the other hand we have to consider that late effects are mainly vascular mediate while the acute ones are due to mitotic dead of replicating cells and the late damage can also happen in absence of acute side effects. Thus, a prolonged follow-up is needed to assess a good long term tolerability of the treatment and it will be the objective of our future analysis.

Although extremely preliminary, it is interesting that a significant fraction of patients showed remission already at two months, suggesting some interplay between high dose per pulse of FFF beams and treatment efficacy. Early local control was achieved in 89% of the cases evaluated. It will be therefore important to perform dedicated studies and to carefully follow patients to assess if there is a radiobiological impact. It was established that the effects of sublethal damage, progression in cell cycle, and repopulation on survival rate, according to dose rate and the biological effects of radiation decreases as the dose rate decreases. Concerning radiation doses delivered with high dose rates, brachytherapy has been historically used safely and with efficacy in various districts. In fact, modern remote afterloader systems can deliver instantaneous dose rates as high as 0.12 Gy/sec (430 Gy/h) at a distance of 1 cm, resulting in treatment times of a few minutes.

Although this wasn't the end point of the current study and longer follow up is needed to evaluate late toxicity and clinical definitive response, this high early response was not observed in previous investigations and might be important, if confirmed, to correlate it to the high dose intensity per pulse of FFF beams.

## Conclusion

SBRT with FFF beams showed, under the acute toxicity profile, to be a safe and feasible technique in 70 consecutive patients with various primary and metastatic lesions in the body. Initial clinical outcomes, in terms of local control are promising. However in further research it is necessary to assess definitive late toxicity and definitive tumor control outcome.

## Competing interests

Luca Cozzi is Head of Research at Oncology Institute of Southern Switzerland and acts as Scientific Advisor to Varian Medical Systems. The authors Marta Scorsetti, Filippo Alongi, Simona Castiglioni, Alessandro Clivio, Antonella Fogliata, Francesca Lobefalo, Pietro Mancosu, Pierina Navarria, Valentina Palumbo, Chiara Pellegrini, Sara Pentimalli, Giacomo Reggiori, Anna M Ascolese, Antonella Roggio, Stefano Arcangeli, Angelo Tozzi and Eugenio Vanetti declare that they have no competing interests.

## Authors' contributions

MA, FA, AF, PM, LC, PN carried out the data, participated in the data evaluation and drafted the manuscript. MA, FA and LC participated in the design of the study and PM and LC performed the statistical analysis. SC, AC, FL, VP,CP, SP, GR, SA, AT, AA carried out the patients record evaluation and followed patients and treatments. The definitive supervision of the paper was done by MA and LC. All authors read and approved the final manuscript.
